# A Novel Experimental Model to Determine the Axon-Promoting Effects of Grafted Cells After Peripheral Nerve Injury

**DOI:** 10.3389/fncel.2019.00280

**Published:** 2019-06-28

**Authors:** Takeshi Endo, Ken Kadoya, Yuki Suzuki, Daisuke Kawamura, Norimasa Iwasaki

**Affiliations:** Department of Orthopaedic Surgery, Faculty of Medicine and Graduate School of Medicine, Hokkaido University, Sapporo, Japan

**Keywords:** peripheral nerve injury, axon regeneration, experimental model, Schwann cell, bone marrow stromal cell

## Abstract

Although peripheral nerves can regenerate, clinical outcomes after peripheral nerve injuries are not always satisfactory, especially in cases of severe or proximal injuries. Further, autologous nerve grafting remains the gold standard for the reconstruction of peripheral nerves, although this method is still accompanied by issues of donor-site morbidity and limited supply. Cell therapy is a potential approach to overcome these issues. However, the optimal cell type for promoting axon regeneration remains unknown. Here, we report a novel experimental model dedicated to elucidation of the axon-promoting effects of candidate cell types using simple and standardized techniques. This model uses rat sciatic nerves and consists of a 25 mm-long acellular region and a crush site at each end. The acellular region was made by repeated freeze/thaw procedures with liquid nitrogen. Importantly, the new model does not require microsurgical procedures, which are technically demanding and greatly affect axon regeneration. To test the actual utility of this model, red fluorescent protein-expressing syngeneic Schwann cells (SCs), marrow stromal cells, or fibroblasts were grafted into the acellular area, followed by perfusion of the rat 2 weeks later. All types of grafted cells survived well. Quantification of regenerating axons demonstrated that SCs, but not the other cell types, promoted axon regeneration with minimum variability. Thus, this model is useful for differentiating the effects of various grafted cell types in axon regeneration. Interestingly, regardless of the grafted cell type, host SCs migrated into the acellular area, and the extent of axon regeneration was strongly correlated with the number of SCs. Moreover, all regenerating axons were closely associated with SCs. These findings suggest a critical role for SCs in peripheral nerve axon regeneration. Collectively, this novel experimental model is useful for elucidating the axon-promoting effects of grafted cells and for analyzing the biology of peripheral nerve axon regeneration.

## Introduction

Although peripheral nerves can regenerate, clinical outcomes after peripheral nerve injuries are not always satisfactory, especially in severe cases and proximal injury cases (Seddon, 1947; Seddon, 1963; Evans et al., 1994; Lundborg, 2000). Further, for reconstruction following peripheral nerve injury, autologous nerve grafting (ANG) has remained the gold standard for more than 7 decades (Seddon, 1947; Millesi, 1973; Evans et al., 1994; Lundborg, 2000; Isaacs, 2010; Daly et al., 2012). However, ANG is still accompanied by issues of donor-site morbidity and limited supply (Isaacs, 2010; Daly et al., 2012). Therefore, a novel therapy promoting peripheral nerve regeneration needs to be developed. Cell therapy is a potential option, according to recent advancements in regenerative medicine (Behfar et al., 2014; Hundepool et al., 2014; Kotton and Morrisey, 2014; Bussolati and Camussi, 2015; Grayson et al., 2015; Sakai and Andersson, 2015; Bitar and Zakhem, 2016; Kadoya et al., 2016; Assinck et al., 2017; Ellis et al., 2017; Chichagova et al., 2018). However, the most effective cell type for induction of peripheral nerve regeneration when grafting into an injured nerve or when combined with a scaffold remains to be determined.

In previous studies that investigated the effect of cell grafts on axon regeneration in the peripheral nervous system (PNS), the peripheral nerve was reconstructed with acellular nerve grafting or an artificial scaffold into which cells were grafted (Guenard et al., 1992; Levi et al., 1994; Rodriguez et al., 2000; Aszmann et al., 2008; Jesuraj et al., 2011; Moore et al., 2011; Jesuraj et al., 2014; Gordon and Borschel, 2017). Because these models require suturing of nerve stumps, axon regeneration sometimes varies, for at least two reasons. One is the difficulty in matching the topography of transected nerve bundles, resulting in poor axon regeneration (Yan et al., 2017). The other is the dependence of axon regeneration on the surgeon’s microsurgical skill (Whitlock et al., 2010). Therefore, an experimental model that does not require a nerve suture procedure is desirable for assessing peripheral nerve axon regeneration. Furthermore, to objectively elucidate the effect of grafted cells on axon regeneration, all other conditions except the graft cell type must be uniform. Here, we report a novel experimental model dedicated to elucidation of the axon-promoting effects of grafted cells in the PNS using simple and standardized techniques.

## Materials and Methods

### Animals

Adult LEWIS rats (Wild-type, Charles River Laboratories Japan, Inc.) were used in all experiments. Their body weight ranged from 160 to 220 g with an average of 195 g. Graft cells were prepared from syngeneic adult LEW-Tg (Gt(ROSA)26Sor-DsRed^*^)7Jmck rats that ubiquitously express the DsRed monomer driven by the gene trap ROSA 26 promoter, supplied by the National BioResource Project (Kyoto University, Kyoto, Japan). The study protocol was approved by the local ethical committee of Hokkaido University. Animals had free access to food and water throughout the study. For animal anesthesia, a mixture of ketamine (75–100 mg/kg, KETALAR^®^, Daiichi Sankyo Propharma Corporation, Tokyo, Japan) and medetomidine (0.5 mg/kg, DOMITOR^®^, Orion Corporation, Espoo, Finland) was administered by intraperitoneal injection.

### Surgical Procedures

**FIGURE 1:**
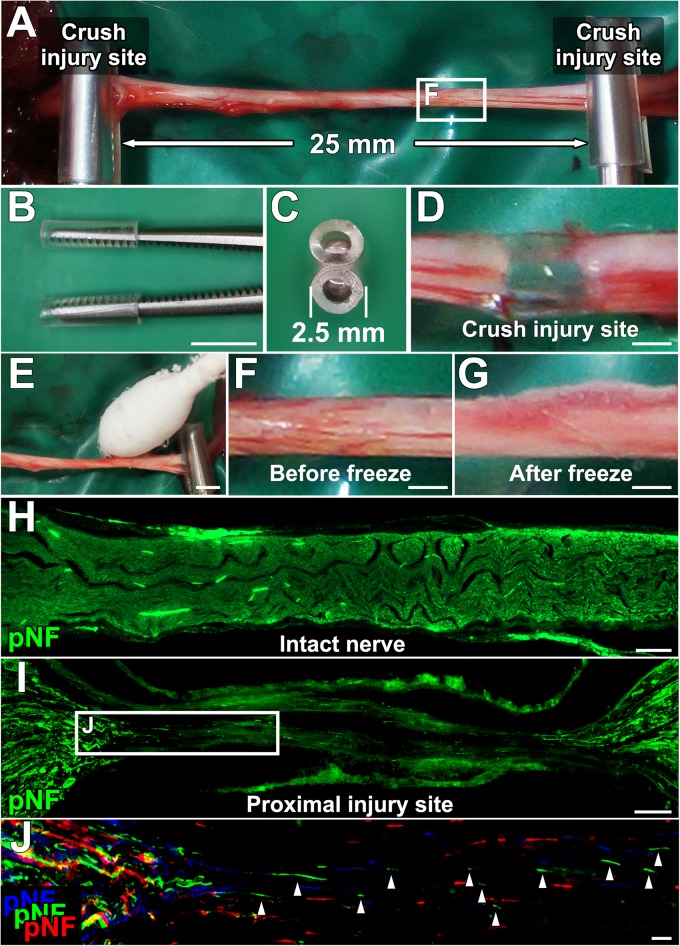
Procedures for establishing a new experimental model. **(A)** The sciatic nerve is injured with a micro-mosquito forceps at two points 25 mm apart. **(B,C)** Lateral and axial views of the micro-mosquito forceps. The tips are covered with a PVC tube. **(D)** Gross appearance of the injury site. **(E)** A cotton swab wet with liquid nitrogen is applied to the nerve. **(F,G)** Gross appearance of the sciatic nerve before and just after the freeze procedure. **(H)** pNF immunolabeled longitudinal section of the intact sciatic nerve. **(I)** Longitudinal section of the proximal injury site just after injury and the FT procedure. **(J)** High magnification image of the boxed area in **I**. Image was generated by stack of 3 serial sections. pNF axons of each section were pseudo colored by blue (medial), green (middle), and red (lateral). No axons present a continuous line, indicating all axons were fragmented. Left is proximal. Scale bars: 5 mm **(B)**, 500 μm **(D)**, 2 mm **(E)**, 500 μm **(F,G)**, 200 μm **(H)**, 100 μm **(I)**, and 10 μm **(J)**.

Anesthetized rats were placed in a prone position, and a longitudinal skin incision (4 cm long) was made from the buttock to distal thigh to expose the entire sciatic nerve. Two crush injuries were made with micro-mosquito forceps (Fine Science Tools, No.13010-12) (Figures 1A–D). The proximal injury was made just distal to the sciatic notch, and the next injury was 25 mm distal to the proximal injury site. To establish the acellular area, this 25-mm long area between the two injury sites was frozen by gentle tapping of a cotton swab wet with liquid nitrogen, followed by spontaneous thawing at room temperature (Figures 1E–G). During this procedure, the micro-mosquito forceps at the injury sites were kept closed. The duration of the forceps closure was always 5 min regardless of the number of freeze and thaw (FT) procedures. The tips of the forceps were covered with a polyvinyl chloride extension tube (1 mm thick, TOP, Tokyo, Japan) to prevent unnecessary cold damage to off-target areas. After this FT procedure, injury sites were marked with a very loose 10-0 nylon stitch of the epineurium. To determine whether the combination of the crush injuries and the FT procedures creates a complete axonal injury and defined acellular region, a total of 24 rat sciatic nerves were divided into the following four groups, (1) injuries alone (*n* = 9), (2) injuries and one cycle of FT (*n* = 5), (3) injuries and five cycles of FT (*n* = 5), and (4) injuries and 10 cycles of FT (*n* = 5).

To clarify the effect of decellularization (DCL) by the FT procedures on axon regeneration in crush or ANG models, 20 animals receiving sciatic nerve lesions were divided into four groups, (1) Injuries alone, (2) Injuries with DCL, (3) ANG alone, and (4) ANG with DCL (five rats per group). ANG implantation was performed by cutting a 25-mm long sciatic nerve and suturing both ends with a 10-0 nylon stich. For DCL, dissected 25-mm long nerves were subjected to five cycles of the FT procedure with liquid nitrogen. Rats were perfused 2 weeks after surgical procedures. To assess long-term axonal growth after ANG, additional two rats received ANG and were perfused 8 weeks later.

### Quantification of Dead Cells

**FIGURE 2:**
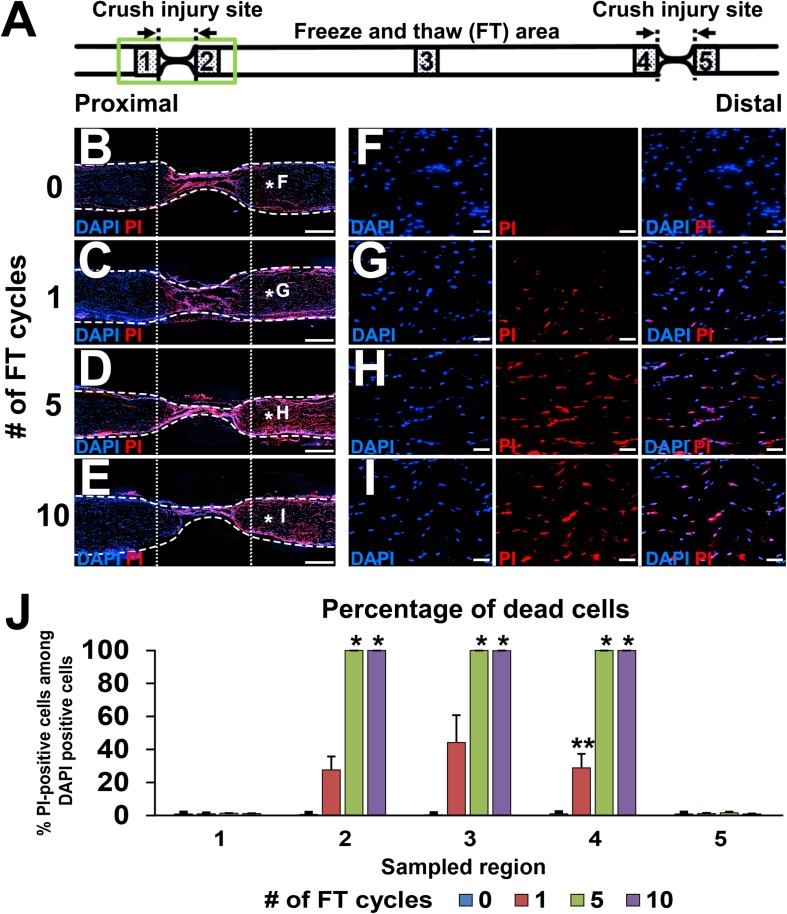
Defined acellular area. **(A)** Illustration indicates the locations of images and quantifications below. **(B–I)** DAPI/PI images of longitudinal sections of sciatic nerves after 0 to 10 cycles of the FT procedures. As the number of cycles of the FT procedures increases, the number of dead cells identified with PI increases. **(B–E)** Low magnification images of the proximal injury site (green boxed area in **A**). Dotted line indicates borders of the injured and uninjured sites. Dashed line indicates the epineurium. **(F–I)** High magnification images of the areas treated with the FT procedures (asterisks in **B–E**). **(J)** Quantification of the percent of dead cells in the five areas shown in **A**. Five and 10 cycles of the FT procedure generate complete cell death in the affected area. ^*^P < 0.05 vs. 0 and one cycle, ^∗∗^*P* < 0.05 vs. other groups. Kruskal–Wallis ANOVA with Steel–Dwass test. Error bars represent the SEM. Scale bars: 500 μm **(B–E)**, 50 μm **(F–I)**.

Immediately after injuries and the FT procedures, sciatic nerves were dissected, and the epineurium was removed. Then, nerves were soaked in propidium iodide (PI) solution (1:1000 in phosphate-buffered saline (PBS), Dojindo, Kumamoto, Japan) for 1 min to stain dead cells, washed with PBS three times, and fixed with 4% paraformaldehyde (PFA) overnight. The next day, nerves were transferred into 30% sucrose solution in 0.1 M phosphate buffer (PB) for at least 24 h. Nerves were longitudinally sectioned at 10 μm thickness with a cryostat (Leica Biosystems CM3050, Wetzlar, Germany) and subsequently covered with cover glass and Mowiol mounting media (Sigma-Aldrich, St. Louis, MO, United States) containing DAPI (4′,6-diamidino-2-phenylindole, 1:1000, Sigma Aldrich). Images of the five areas shown in Figures 2B–I were taken at 200× magnification using a regular fluorescent microscope system (Olympus BX53, Tokyo, Japan). Because we noticed that the PI signal faded gradually after exposure to Mowiol, images were taken immediately after placing a cover glass on the slide. The numbers of PI- and DAPI-positive cells were manually counted from these images.

### Cell Preparation

All cells used in this study were prepared from transgenic adult LEWIS rats that ubiquitously expressed red fluorescent protein (RFP) and that were syngeneic to wild-type LEWIS rats. Cell viability, which was assessed with trypan blue (Life Technologies, Grand Island, NY, United States), was within the range of 92–95% for all graft cell preparations. Cell populations were assessed by immunostaining.

### Schwann Cells (SCs)

Schwann cells were harvested according to modification of a previous protocol (Kaewkhaw et al., 2012). Sciatic nerves running from the sciatic notch to the end of the femur were dissected, cut into 1- to 2-mm pieces using micro-scissors after removal of the epineurium, and transferred to enzymatic digestion medium containing 1% collagenase I (Sigma-Aldrich) and 0.125% trypsin in Dulbecco’s modified Eagle medium (DMEM)/Ham’s F-12 (Wako, Osaka, Japan). After incubation for 1 h at 37°C, tissues were mechanically dissociated by pipetting 30 times in 1 ml medium, consisting of DMEM/Ham’s F-12 supplemented with 10% fetal bovine serum (FBS), 1% GlutaMAX (Thermo Fisher Scientific, Waltham, MA), and 1% penicillin-streptomycin (PS, Thermo Fisher Scientific, United States). Then, dissociated cells were seeded in 75-cm^2^ flasks (1.0 × 10^5^ cells/mm^2^) coated with poly-L-lysine (Sigma-Aldrich) and laminin (Sigma-Aldrich) for 2 days.

### Marrow Stromal Cells (MSCs)

Bilateral femurs and tibias were dissected, and bone marrow was flushed with 5 ml DMEM supplemented with 20% FBS, 1% GlutaMAX, and 1% PS. Then, the cell suspension was triturated 10 times through an 18-gauge needle to shear the connective tissue, seeded in 75-cm^2^ flasks, and incubated for 4 h, followed by medium replacement to remove floating cells and debris. Culture medium was changed every 3 days during incubation, and adherent cells were expanded for three passages.

### Fibroblasts

Skin was dissected from the rat’s back and cut into pieces of 0.25–0.30 cm^2^ after subcutaneous tissue was shaved off. Three to four skin pieces were placed into six-well plates with DMEM supplemented with 10% FBS, 1% GlutaMAX, and 1% PS and gently pressed with a sterile cover glass so that the skin pieces attached to the bottom of the plate. One week later, proliferating cells were detached using 0.25% trypsin and reseeded into 75-cm^2^ flasks for further incubation. Medium was changed every 3 days during incubation, and adherent cells were expanded for three passages.

### Cell Grafting

A total of 30 rat sciatic nerves were divided into the following five groups, (1) SC grafts, (2) MSC grafts, (3) fibroblast grafts, (4) no cell grafts, and (5) injury alone (*n* = 6 per group). In the cell graft groups, 2 million cells in 10 μl PBS were grafted into the acellular region by four injections with a 34-gauge needle and a NanoFil syringe (World Precision Instruments, Sarasota, FL, United States), and 10 μl PBS alone was injected in the other two groups. To achieve a uniform distribution of grafted cells, the tip of the needle was placed at the center of the proximal and distal halves of the acellular region as described in Figure 5C on both sides of the sciatic nerve.

### Immunohistochemistry and Immunocytochemistry

For immunohistochemistry, sciatic nerves were harvested after perfusion with 4% PFA in 0.1M PB, followed by overnight fixation with 4% PFA at 4°C. On the following day, nerves were transferred into 30% sucrose in 0.1M PB and stored until sectioning. Nerves were longitudinally sectioned on a cryostat at 10-μm intervals and directly mounted on 10 slides in order. To confirm lesion completeness, 4 sciatic nerves immediately after receiving injuries and 5 cycles of the FT procedures were serially mounted on 10 slides. Total of four sections were mounted onto each slide. For immunocytochemistry, cultured cells were fixed with 4% PFA in 0.1M PB for 15 min. Fixed cells or sections were incubated overnight with primary antibodies against RFP (1:200, goat from Sicgen, Portugal), pan neurofilament (pNF, 1:1000, mouse from BioLegend, San Diego, CA, United States), S100β (1:200, rabbit from Abcam, Cambridge, United Kingdom), and CD90 (1:100, mouse from Bio-Rad, Hercules, CA, United States) at 4°C. Then, after washing with Tris-buffered saline, sections were incubated in Alexa 488 conjugated donkey anti-mouse, anti-rabbit, Alexa 594 conjugated donkey anti-rabbit, anti-goat, Alexa 647 conjugated donkey anti-mouse secondary antibodies (1:1000, Jackson ImmunoResearch, West Grove, PA, United States) and DAPI for 1 h at room temperature.

### Quantification

Three consecutive sections on the same slide from the middle part of the nerve were used for axon and SC quantification. Lines perpendicular to sections were set at points 7.5, 15, 22.5, 30, and 40 mm distal and 1.5 mm proximal to the injury site. The numbers of pNF-labeled axons crossing each line were quantified. For normalization, the sum of the axon numbers of three sections was divided by the sum of the length of each line as an axon density. To calculate the percentage of axon regeneration, the axon density of each point was divided by the density at the uninjured site, which was 1.5 mm proximal to the injury site.

For quantification of the amount of SCs, the S100β immunoreactive area was measured with ImageJ software (Schneider et al., 2012) in a 100-μm wide region at five points, which were 0, 7.5, 15, 22.5, and 30 mm distal to the proximal injury site. Then, the sum of the S100β immunoreactive area of three sections was divided by the total of the quantified area to calculate the percentage of the S100β immunoreactive area.

To evaluate the close association between axons and SCs, sections triple stained for S100β, pNF, and DAPI were imaged with confocal laser microscopy (Zeiss LSM780, Oberkochen, Germany). At least 30 regenerating axons per rat were randomly selected and photographed at 600× magnification to determine the three-dimensional localization of SCs and regenerating axons.

### Statistical Analysis

Normality of the data distribution was assessed with the Shapiro–Wilk test. Kruskal–Wallis analysis of variance (ANOVA) with the Steel–Dwass test was used for dead cell quantification in the nerve treated with the FT procedure. For other statistical analyses, multiple-group comparisons were made with one-way ANOVA and the Tukey–Kramer test, and two-group comparisons were made with the unpaired two-tailed Student’s *t*-test. All analyses were performed with JMP software (SAS, Cary, NC, United States) with a pre-specified significance level of 95%. Data are presented as the mean ± standard error of the mean (SEM).

## Results

### A Novel Experimental Model Consists of Two Crush Injuries With Freeze and Thaw Procedures

We specified six requirements for an experimental model that can elucidate the effect of test cells on peripheral nerve axon regeneration. They were (1) no nerve suture procedure, (2) defined acellular region, (3) no spared axons, (4) capability of having a cell composition similar to that of ANG as a positive control, (5) completely equal conditions except for the test cell type among groups, and (6) sufficient range between positive and negative controls for clear detection of axon-promoting effects. To meet these criteria, we employed a DCL procedure and two crush injuries. The sciatic nerve of an adult Lewis rat was crushed in two places at a 25-mm interval with micro-mosquito forceps (Figures 1A–D). For DCL, this 25-mm long region underwent FT using liquid nitrogen (Figures 1E–G).

Lesion completeness was investigated with serial sections of sciatic nerves fixed immediately after crush injuries and the FT procedures. At the crush injury site, most of pNF immunoreactivity disappeared, and all of residual pNF labeled axons were fragmented (Figures 1H–J), indicating that all axons were lesioned. To label dead cells following the FT procedure, sciatic nerves were incubated with PI for 1 min immediately after the FT procedures and then fixed in PFA. A single cycle of the FT procedure induced death of 28–44% of cells in the affected region (Areas 2–4 in Figure 2A), whereas almost all cells were dead after more than five cycles of the FT procedure (Figures 2A–J). In the untreated regions (Areas 1 and 5 in Figure 2A), very few cells were detected as dead in all tested groups, indicating that the FT procedure did not affect neighboring regions. These findings indicate that five cycles of the FT procedure in combination with crush injuries are sufficient to create a defined acellular region with complete axonal injuries, satisfying the first three requirements of the model.

### Crush With DCL Shows Sufficient Range to Detect Axon Regeneration

**FIGURE 3:**
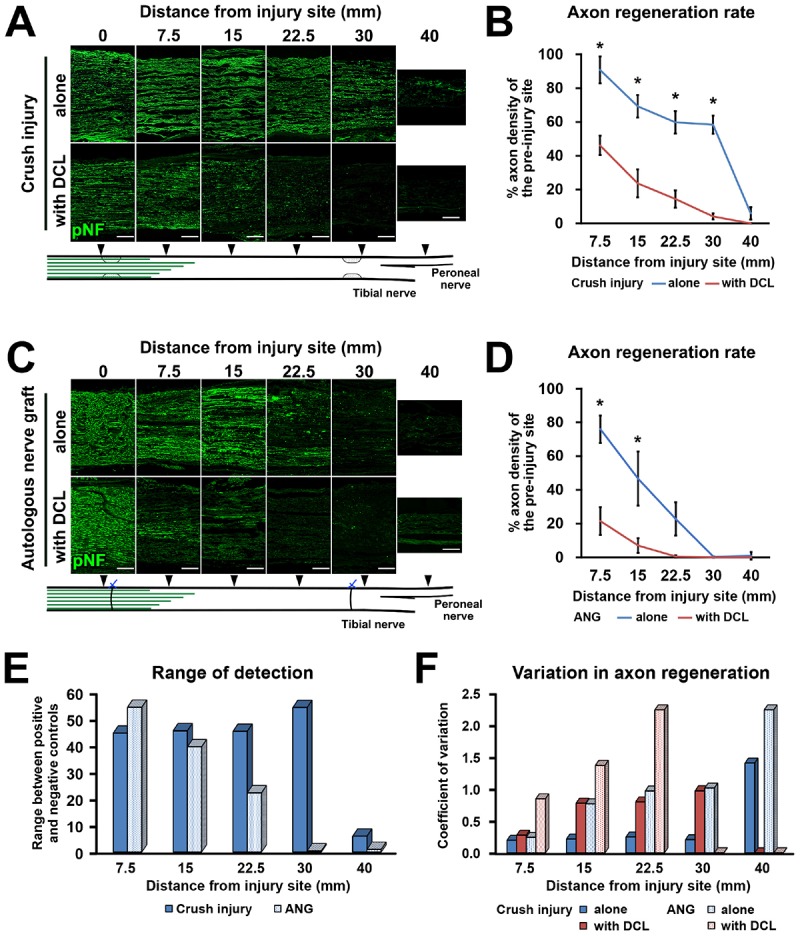
High range for detection of axon regeneration. **(A)** Representative images of longitudinal sections immunolabeled for axons (pNF) at the points of 0, 7.5, 15, 22.5, 30, and 40 mm distal to the proximal injury site 2 weeks after crush injury with or without DCL. Left is proximal. **(B)** Quantification of the axon regeneration rate in the crush injury model. ^*^P < 0.05; Student’s t test. Error bars represent the SEM. **(C)** Representative images of longitudinal sections 2 weeks after ANG implantation with or without DCL. Left is proximal. **(D)** Quantification of the axon regeneration rate in the ANG model. ^*^P < 0.05; Student’s *t*-test. Error bars represent the SEM. **(E)** Range to detect axon regeneration in both the crush injury and ANG models. **(F)** Variation in quantified axon regeneration. Scale bars: 200 μm **(A,C)**.

**FIGURE 4:**
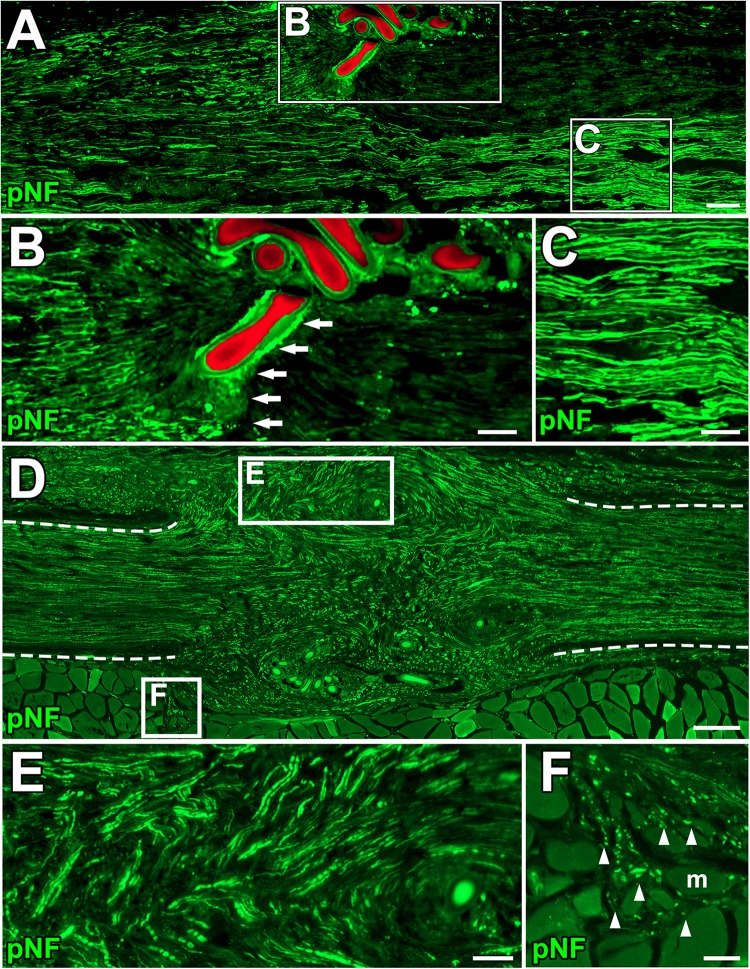
Blockade of axonal growth in the ANG model. **(A)** Longitudinal section of sciatic nerve 2 weeks after ANG. The suture blade (pseudo colored with red) interferes with axon regeneration. Left is proximal. **(B,C)** High magnification images of the boxed areas in **A**. Arrows indicate axons physically blocked by the misplaced suture blade. **(D–F)** Longitudinal sections of the sciatic nerve 8 weeks after ANG. **(D)** Axons regenerated into not only the distal nerve but also ectopic locations from the gap between nerve stumps. Dashed lines indicate the epineurium. **(E)** High magnification image of the boxed area in **D**. Axons deviated from the original pathway and grew out. **(F)** High magnification image of the boxed area in **D**. Axons grew into muscle fibers. Arrowheads indicate individual axons. m, muscle fiber. Scale bars: 100 μm **(A)**, 50 μm **(B,C,E,F)**, 200 μm **(E)**.

Next, we clarified how DCL of a 25-mm long region impaired axon regeneration after peripheral nerve injury. In other words, we determined whether the new model had a sufficient range between positive and negative controls for clear detection of axon-promoting effects. Rats received either crush injuries alone (positive control) or crush injuries with DCL (negative control), followed by perfusion 2 weeks later. In addition, groups with ANG with or without DCL were added for comparison. Quantification of immunostained axons revealed that DCL significantly reduced axon regeneration compared to the no DCL groups in both models (Figures 3A–D), indicating the critical role of residual cells in axon regeneration as reported previously (Ide et al., 1983; Hall, 1986a, 1986b; Gulati, 1988). The new model showed significant differences between with and without DCL at four distances from the injury site: 7.5, 15, 22.5, and 30 mm. At the 30-mm point, rats with crush injuries alone had 60% regenerating axons, whereas rats with injuries and DCL rarely showed axon regeneration, indicating that the range to detect the effect of candidate cells on axon regeneration is mainly from 0 to 60%. In addition, if a graft of test cells showed axon regeneration more than 60%, these cells may be more effective than ANG. On the contrary, the ANG model showed significant differences only at 7.5 and 15 mm from the injury site. Further, the range for the ANG model between with and without DCL was smaller than that of the crush model (Figure 3E). Moreover, the quantity of axon regeneration varied more in the ANG model than the new model (Figure 3F). This reduced and heterogeneous axon regeneration in the ANG model was at least partially due to the occasional blockade of axonal growth by the suture blade (Figures 4A–C) and ectopic axon regeneration or sprouting into surrounding non-nervous tissue from incomplete sealing of the epineurium (Figures 4D–F). Collectively, these findings indicate that the new model satisfies the last three requirements we specified and is more suitable for assessing the effect of cell grafting than the ANG model which is one of the conventional experimental models to assess the effects of cell grafts.

### SC Grafts Promote Axon Regeneration, Whereas MSC Grafts Do Not

**FIGURE 5:**
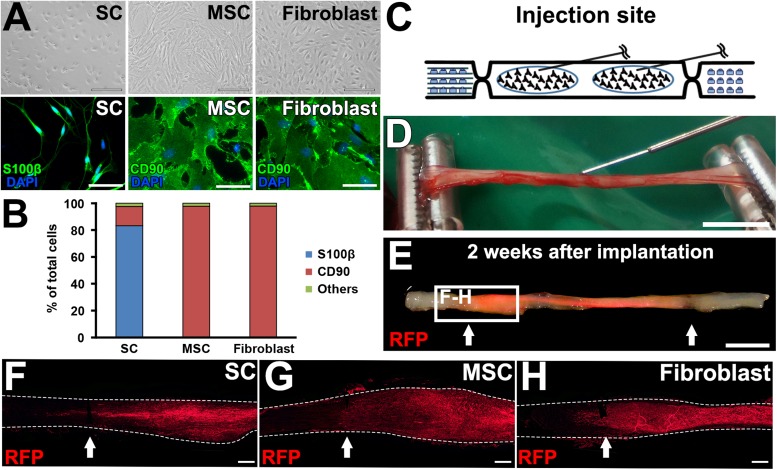
Graft cell survival. **(A)** Phase contrast and immunofluorescence images of cultured cells. **(B)** Purity of cultured cells. **(C)** Schematic of the cell graft procedure. **(D)** Actual photo of injection of cells. **(E)** When a fluorescent light was applied, the RFP signal was detected in the fixed sciatic nerve that was injected with RFP-expressing SCs. Arrows indicate the injury sites. **(F–H)**, Longitudinal sections around the proximal injury sites show the distribution of grafted cells. Arrows indicate the injury sites. Dashed lines indicate the outline of nerves. Scale bar: 100 μm (**A**, top), 50 μm (**A**, bottom), 5 mm **(D)**, 5 mm **(E)**, 500 μm **(F–H)**.

**FIGURE 6:**
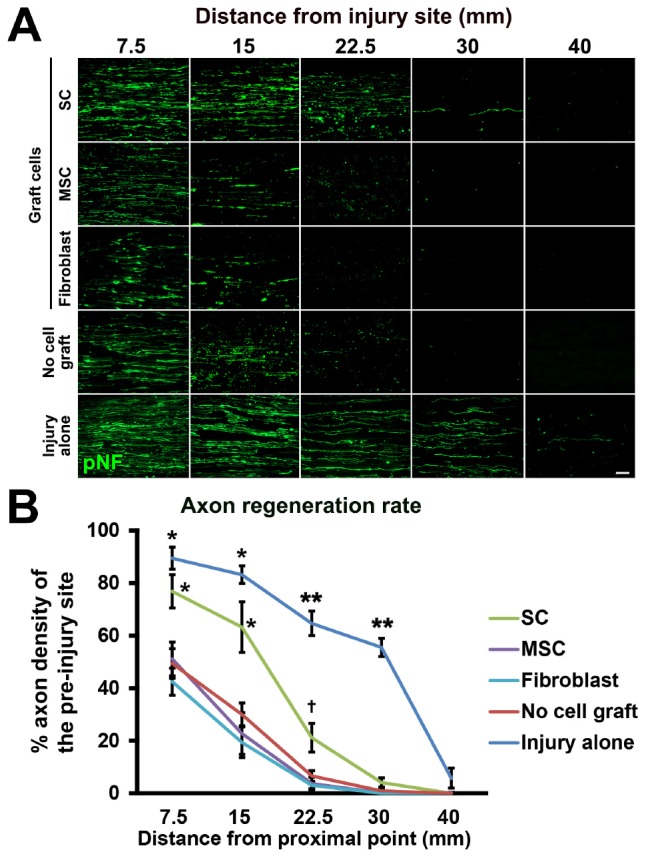
Accelerated axon regeneration by SCs. **(A)** Representative images of regenerating axons labeled by pNF in each group. Left is proximal. **(B)** Quantification of regenerating axons. ^*^P < 0.05 vs. MSCs, fibroblasts, and no cell graft, ^∗∗^*P* < 0.05 vs. SCs, MSCs, fibroblasts, and no cell graft, ^†^*P* < 0.05 vs. MSCs and fibroblasts. One-way ANOVA with the Tukey–Kramer test. Error bars represent the SEM. Scale bar: 50 μm **(A)**.

Next, we tested the actual utility of this model by grafting several cell types. We tested SCs (Aszmann et al., 2008; Jesuraj et al., 2014) and MSCs (Dezawa et al., 2001; Tohill et al., 2004; Chen et al., 2007; Wang et al., 2008; Karaoz et al., 2009; Ladak et al., 2011; Pan and Cai, 2012; Hundepool et al., 2014; Wakao et al., 2014). As a control, fibroblasts and vehicle injections were also tested (Figure 5A). The purities of the prepared cells were 83, 98, and 98% for SCs, MSCs, and fibroblasts, respectively (Figure 5B). A total of 2 million RFP-expressing cells in 10 μl PBS were injected into the acellular region (Figures 5C,D) at the same time of the injury and DCL procedure. Two weeks after cell grafts and injuries, rats were perfused, followed by histological assessment. Macroscopically, all types of grafted cells survived well and filled the acellular region (Figure 5E). Immunolabeling of RFP cells showed that grafted cells were well distributed throughout the acellular region, and few cells migrated past the crush sites (Figures 5F–H). Regarding axon regeneration, rats with injury alone showed the best regeneration as expected (Figures 6A,B). Next was the SC graft, which induced significantly more axon regeneration than the other groups except injury alone at three points from 7.5 to 22.5 mm from the proximal injury site (Figure 6B). On the other hand, the MSC graft did not demonstrate any axon-promoting effect compared to negative controls. These results indicate that the new model can clearly differentiate effects of cell grafts on axon regeneration and that grafts of SCs but not MSCs accelerate axon regeneration.

### The Amount of SCs Strongly Correlates With Axon Regeneration

**FIGURE 7:**
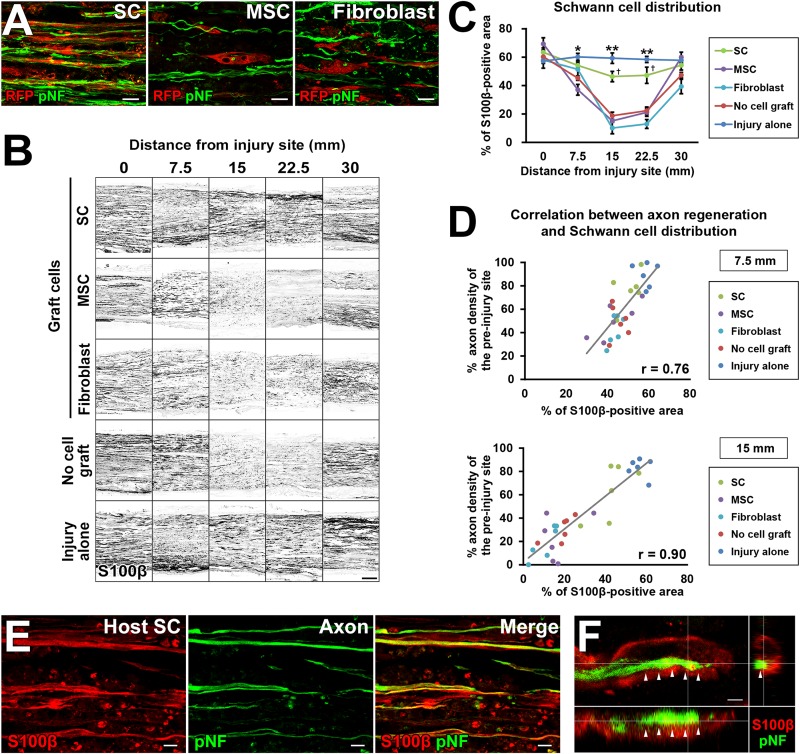
The critical role of SCs in axon regeneration. **(A)** Double immunolabeling of grafted cells (RFP) and regenerating axons (pNF). Grafted SCs are closely associated with regenerating axons, whereas MSCs and fibroblasts are not. **(B)** Both graft- and host-derived SCs are visualized with S100β immunolabeling. Representative images from longitudinal sections of all groups. The SC graft group and injury alone group show dense S100β immunoreactivity in the middle of the nerve. **(C)** Quantification of SC distribution. ^*^P < 0.05 vs. MSCs, fibroblasts, and no cell graft, ^∗∗^*P* < 0.05 vs. SCs, MSCs, fibroblasts, and no cell graft, ^†^*P* < 0.05 vs. MSCs, fibroblasts, and no cell graft; one-way ANOVA with the Tukey–Kramer test. Error bars represent the SEM. **(D)** The correlation between the extent of axon regeneration and the amount of SCs. Each color represents the type of cells grafted. Gray lines indicate regression lines. The Pearson correlation coefficients are 0.76 and 0.9 at the 7.5-mm and 15-mm points distal to the proximal injury sites, respectively. **(E)** Double immunolabeling of host SCs (S100β) and regenerating axons (pNF) in the no cell graft group at 7.5 mm distal to the proximal injury sites. Host SCs are closely associated with regenerating axons. **(F)** Three-dimensional confocal microscopic image of the no cell graft group demonstrates that the tip of a regenerating axon, which shows a growth cone-like morphology, is closely associated with a SC. Arrows indicate the border between the axon and SC. Scale bar: 10 μm **(A)**, 200 μm **(B)**, 10 μm **(E)**, and 2 μm **(F)**.

To explore the mechanism of how grafted cells support axonal growth, we analyzed positional relationships between RFP-expressing graft cells and regenerating axons. The grafted SCs had a very close association with regenerating axons (Figure 7A), which is similar to the finding of an “axon-SC partnership” described previously (Chen et al., 2005; McDonald et al., 2006), implying that SCs promote axonal growth via a contact-mediated mechanism. On the contrary, MSCs and fibroblasts were not located close to axons at all (Figure 7A), indicating that their effects on axons, if any, are not mediated by direct contact with axons. Because host SCs migrate and proliferate (Hall, 1986a; Ide et al., 1990), we investigated all SCs from both the host and graft by immunolabeling with the pan-SC marker, S100β. As expected, SCs were observed in the acellular region in all groups (Figure 7B). Quantification of the S100β-positive area revealed that the injury alone (no DCL) group showed the greatest SC area among all groups (Figure 7C). The SC graft group was the second, exhibiting more SCs than any other groups except the injury alone group (Figure 7C). Neither the MSC nor the fibroblast graft group showed an increase in the S100β area compared to the no cell graft group, indicating that these cells did not promote migration or proliferation of host SCs, and also did not differentiate into SCs. Next, we analyzed the correlation between the amount of SCs and the extent of axon regeneration. The correlation coefficients were 0.76 and 0.9 at the 7.5- and 15-mm points from the proximal injury site, respectively (Figure 7D), indicating that the amount of SCs was strongly correlated with axon regeneration. Further, detailed examination with confocal microscopy showed that all examined regenerating axons in all groups were closely associated with SCs (Figure 7E). When focusing on the tip of a regenerating axon identified by growth cone-like morphology, a SC preceded it and was also closely associated with it (Figure 7F). These findings strongly indicate that SCs play a critical role in PNS axon regeneration and that this effect may be mediated partially by direct contact between axons and SCs.

## Discussion

Here, we developed a new experimental model for the study of cell therapy for PNS regeneration. To the best of our knowledge, this is the first model dedicated to assessing the efficacy of grafted cells on axon regeneration. Because this model does not require a suture procedure, the topography of nerve bundles is maintained, axons do not grow out of nerves, and axon regeneration is independent of surgical skills, resulting in a reliable and easily established model. Moreover, with DCL of a 25-mm long region, the model has a sufficient range of detection of the axon-promoting effects. A comparative study showed a greater range for detection of axon-promoting effects and less variability in quantification of axon regeneration, compared to the ANG model. The effect of the SC graft on axon regeneration was clearly identified at three points from 7.5 to 22.5 mm from the proximal injury site, indicating superior utility of this model. Importantly, this model does not require special instruments, reagents, or techniques to perform DCL of a defined region. Therefore, this model can be established in any laboratory without requiring additional time or costs.

By grafting different types of cells into this model, two important findings were observed about the role of SCs in axon regeneration in the PNS. First, the extent of axon regeneration is strongly correlated with the number of SCs. This observation is independent of administration of the cell graft, types of grafted cells, and types of host- or graft-derived SCs. Although previous studies reported the importance of SCs (Hall, 1986b; Gulati, 1988) in PNS regeneration, as far as we know, this is the first report to show a linear relationship between the number of SCs and the extent of axon regeneration. This raises the possibility that simply increasing the number of SCs will be a simple and potential approach to accelerate axon regeneration in the PNS, although a ceiling effect will be present due to limited space for grafting. Also, an efficient intervention to promote SC migration from the proximal region to the region of axon regeneration may be another potential strategy to improve regeneration.

The second finding is that all regenerating axons were closely associated with SCs. This was previously described as an “axon-SC partnership” (Chen et al., 2005; McDonald et al., 2006). Our data support this idea because we observed that SCs precede and guide the regenerating axon (Figure 7F) via direct contact. These observations may sound contradictory to a relatively old report that showed that PNS axons can regenerate without any contact with SCs (Ramon and Cajal, 1928; Ide et al., 1990). However, the discrepancy can be explained by differences in the locations investigated and time points after injury. Axon growth without SCs was observed very close to the injury site a couple days after injury (Ide et al., 1983; Chen et al., 2005), whereas we found a close association between regenerating axons and SCs 7.5 to 30 mm distal to the injury site 2 weeks after injury. Because SCs change their function according to the time and location after injury (Scheib and Hoke, 2013; Jessen and Mirsky, 2016), this discrepancy appears reasonable. Additionally, we may need to consider the possibility that the detailed mechanism underlying axon regeneration may vary as a function of the distance from the injury site (Fernandes et al., 1999). Collectively, these observations indicate that the new experimental model succeeded in revealing new biology of peripheral nerve axon regeneration.

In the current study, we did not observe any axon-promoting effect of MSCs, whereas many studies have reported regenerative effects of MSCs after peripheral nerve injury (Dezawa et al., 2001; Tohill et al., 2004; Keilhoff et al., 2006; Chen et al., 2007; Shimizu et al., 2007; Wang et al., 2008; Karaoz et al., 2009; Wang et al., 2010; Ladak et al., 2011; Pan and Cai, 2012; Zarbakhsh et al., 2012; Hundepool et al., 2014; Wakao et al., 2014; Carrier-Ruiz et al., 2015). Seven possible reasons may explain this contradictory result: (1) Usage of the term “MSC” has been confusing as described in recent studies (Dominici et al., 2006; Salem and Thiemermann, 2010; Sipp et al., 2018), and only a small proportion of marrow stromal cells (MSCs) are mesenchymal stem cells (Salem and Thiemermann, 2010). Because the current study used “MSCs” but not “mesenchymal stem cells,” the MSCs in the current study may have failed to show a significant effect, unlike the studies using mesenchymal stem cells (Wang et al., 2008; Ladak et al., 2011). (2) Similar to the current study, several studies have reported that a graft of undifferentiated MSCs fails to promote axon regeneration in the PNS (Keilhoff et al., 2006; Shimizu et al., 2007; Ladak et al., 2011), indicating that the effect of a MSC graft in the PNS remains controversial. Similarly, the effect of a MSC graft in spinal cord injury models has not been determined yet (Qu, 2017; Vismara et al., 2017). (3) As a control, an empty tube was used in two studies (Chen et al., 2007; Wang et al., 2010), whereas an acellular nerve was utilized in the current study. It is reasonable to assume that achievement of superiority to an empty tube is easier than to an acellular nerve. (4) The length of the acellular region in the current study was 25 mm, which is much longer than the length in other studies, which are usually from 10 to 15 mm (Hundepool et al., 2014). Our prior experiment demonstrated that an acellular region 15 mm long was substantially filled with host SCs and failed to provide a sufficient range to detect the axon-promoting effect of grafted cells (data not shown), suggesting that the length of the acellular region is important for axon regeneration and host SC migration. (5) Some studies concluded the therapeutic effects of MSCs based on evaluation of myelination, electric nerve conduction velocity, and functional performance, but not axon regeneration (Wang et al., 2010; Zarbakhsh et al., 2012), whereas the current study focused exclusively on axon regeneration. (6) The time points to determine axon regeneration are different. The current study employed a 2-week time point to evaluate speed as well as extent of axon regeneration, but other studies used longer time points from 6 to 12 weeks (Keilhoff et al., 2006; Chen et al., 2007; Wang et al., 2010; Ladak et al., 2011). A longer observation period may eliminate the effect of accelerated axon regeneration observed at an early time point and demonstrate a different result. (7) The number of grafted cells was different among studies. An optimal cell concentration of the MSC graft may be needed to induce maximum therapeutic effects (Raheja et al., 2012). Other important topics about MSC grafts are their effects on host SCs and their differentiation into SC-like cells. Unlike other studies (Wang et al., 2009; Carrier-Ruiz et al., 2015), the current study did not show that a MSC graft promoted migration or proliferation of host SCs, and a very small number of grafted MSCs were S100 immunoreactive (data not shown). These discrepancies may also be due to the same reasons mentioned above. Some previous studies using undifferentiated MSCs (Keilhoff et al., 2006; Shimizu et al., 2007; Ladak et al., 2011) showed similar results as ours.

Because PNS regeneration requires highly orchestrated interactions among multiple cell types and biological phenomena, further studies need to clarify important topics related to this new model. One is the innate inflammatory response, because accumulating evidence suggests that the recruitment and activation of inflammatory cells significantly affect PNS regeneration (Gaudet et al., 2011). The 25 mm-long acellular region may have different inflammatory responses from other models. Also, evaluation of innate inflammatory responses with different cell grafts will provide a deeper understanding of their mechanisms. Another topic for future exploration is basal lamina tubes (BLTs) in which SCs proliferate and axons grow (Scheib and Hoke, 2013). One electron microscopy study reported that the ultra-structure of the peripheral nerve was maintained after the FT procedure (Ide et al., 1983), suggesting the possibility that axons grow in the original BLTs of the acellular region. However, several important questions remain to be answered, including whether the injection of cells alters the ultra-structure of BLTs, whether grafted cells integrate with existing BLTs or form new tubes and Büngner bands (Bungner, 1891), or whether this new model provides better circumstances for axons to reach their original targets with great accuracy or whether the axons grow in a more random manner.

## Conclusion

In conclusion, we developed a novel experimental model especially dedicated to elucidation of the axon-promoting effect of grafted cells in the PNS using simple and standardized techniques. This model clearly demonstrated that a graft of SCs but not MSCs promoted axonal growth after peripheral nerve injury and elucidated the cellular biology of axon regeneration in the PNS.

## Data Availability

The datasets generated for this study are available on request to the corresponding author.

## Ethics Statement

This study was carried out in accordance with the recommendations of the local ethical committee of Hokkaido University. The protocol was approved by the local ethical committee of Hokkaido University.

## Author Contributions

TE, KK, and NI designed the research. TE conducted the research. YS and DK contributed with unpublished reagents and analytic tools. TE and KK analyzed the data. TE and KK wrote the manuscript.

## Conflict of Interest Statement

The authors declare that the research was conducted in the absence of any commercial or financial relationships that could be construed as a potential conflict of interest.
